# Determinants of infant and young child feeding practices in Rupandehi, Nepal

**DOI:** 10.1186/s13104-016-1956-z

**Published:** 2016-03-02

**Authors:** Kapil Prasad Gautam, Mandira Adhikari, Resham Bahadur Khatri, Madhu Dixit Devkota

**Affiliations:** Save the Children, Kathmandu, Nepal; Nepal Development Society, Bharatpur, Nepal; Department of Community Medicine and Public Health, Institute of Medicine, Tribhuvan University, Kathmandu, Nepal

**Keywords:** Acceptable diet, Complementary feeding, Infant and young child feeding, Dietary diversity, Nepal

## Abstract

**Background:**

Undernutrition is a major problem in Nepal and meeting the minimum dietary standard is essential for growth and development of young children. Continuous monitoring of such practices is important to inform policy and program formulation. This study aimed to assess complementary feeding practices, and associated factors in Western Nepal.

**Methods:**

This was a cross-sectional study conducted in Rupandehi district of Western Nepal. Face-to-face interviews were conducted among 178 mothers of young children aged 6–23 months using a structured questionnaire and data on complementary feeding practices. These practices were reported as frequency distribution and the factors associated were ascertained using multiple logistic regression.

**Results:**

Only 57 % of mothers initiated complementary feeding at the age of 6 months. While the proportion of young children receiving minimum meal frequency was reasonably high (84 %), meal diversity (35 %) and minimum acceptable diet (33 %) remained low. Maternal education and having had their children’s growth monitored were independently associated with receiving minimum acceptable diet.

**Conclusion:**

Few infants and young children received the recommended infant and young children feeding practices. Implementing health promotion programs that educate and enhance the skills of mothers should be a priority for future nutrition interventions.

## Background

The time between a child’s birth and 2 years of age is a critical window of opportunity to ensure the child’s development through optimum feeding practices [1]. Even mild or moderate undernutrition during this period can cause irreversible damage [2]. As an infant completes 6 months of age, a mother’s milk is no longer sufficient to fulfil the child’s increasing nutritional need. Suboptimal breastfeeding and poor complementary feeding practices are responsible for under nutrition among young children [3]. Optimal infant and young child feeding can have the potential to prevent an estimated 19 % of all under-five deaths, more than any other single preventive intervention [4]. Therefore, World Health Organisation (WHO) has recommended core indicators for infant and young child feeding (IYCF), of which timely introduction of soft, solid or semi-solid foods, minimum dietary diversity, minimum meal frequency, and minimum acceptable diet are related to late infancy, and thereafter up to 2 years of age [5].

Early introduction of supplementary feeding is a very common cultural practices in the South Asian region including Nepal [6–9] which has a historically high burden of under-nutrition [8, 10]. The Nepal Demographic and Health Survey (NDHS) 2006 reported about 70 % of children aged 6–8 months children were introduced to complementary foods in Nepal [11]. Similarly, the prevalence of minimum meal frequency, minimum dietary diversity, minimum acceptable diet is 82, 34.2 and 32 % respectively [11]. By 2011, children 6–23 months of age were offered minimum dietary diversity (30.4 %), minimum meal frequency (76.6 %), and acceptable diet (26.5 %) [12] showing deteriorating conditions in infant feeding practices. These data suggest a much needed focus on improvement and monitoring of these practices while the interventions are being implemented in Nepal.

Nepal has a high burden of under-nutrition among young children. IYCF practices are to be monitored continuously to provide evidence-based decision-making in interventions designed to reduce under-nutrition in Nepal. Few previous studies have reported on the time of introduction of complementary feeding, meal frequency, meal diversity and acceptable diet. Maternal education has been found to be associated with timely introduction of complementary feeding [13, 14], minimum meal frequency, minimum dietary diversity, and minimum acceptable diet [12, 15]. Other determinants that have been associated with complementary feeding practices are household wealth status, geographical location, exposure to media, maternal age, and the utilization of antenatal and postnatal visits [12, 15–18]. While most of the studies reported based on the national surveys [12, 14, 19], these national reports do not necessarily reflect the every diverse ethnic communities of Nepal [20]. Additional information is needed to provide more evidence to monitor progress at the local level. The current study aimed to measure the prevalence of timely initiation of complementary feeding and minimum acceptable diet, and the factors associated with these infant feeding practices in Western Nepal.

## Methods

### Study setting, sampling and sample size

A cross-sectional study was conducted during August to September 2011 in Padsari Village Development Committee (VDC) of Rupandehi district bordering to India. VDC is the lowest administrative unit of the Government of Nepal. The study area is peri-urban area with diversified culture and ethnicity where majority of population belong to indigenous Tharu groups, Dalits and Janajati groups [20]. As per census 2011, it has population of 7768 living in 1234 households [21]. The study population was mothers and their children aged 6–23 months. Total population of the children aged 6–23 months in Rupdendhi district is 32,876 [22]. The sample size (180) was calculated using the formula provided by Daniel et al. [23]: n = Z^2^pq/L^2^ where, prevalence of inappropriate infant feeding (p) = 0.36 (36 % children age 6–23 months from Western development region are not fed according to recommended IYCF practices [24]); prevalence of appropriate feeding (q) = 0.64; level of significance (α) = 5 %; Z = 1.96; allowable error (L) = 20 % of p = 0.072; non-response rate = 5 %.

**Fig. 1:**
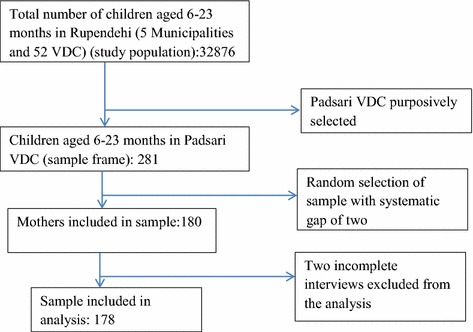
Flow chart of sample selection

The list of the children aged 6–23 months was obtained from a comprehensive list maintained by female community health volunteers (FCHVs) for Baalvita (micronutrient supplementation) program, and children missed from micronutrient program were supplemented by immunization register maintained by the local sub health post. With the existing immunization coverage being more than 95 % continuously for last few years, it is assumed that combination of the list obtained from these two sources would include all of the children (6–23 months) in the study areas. A total of 180 children were selected from the sampling frame of 281 children using systematic random sampling. The mothers of selected children were interviewed visiting their home. First mother was selected randomly and then every third (having gap of two) mothers were interviewed. If mothers were not met during home visits for interview, next visits were conducted to collect the data (Fig. 1). Our inclusion criteria were mothers whose children not sick in the past 24 h. Similarly, exclusion criteria were children with any disabilities or mothers who were not able to speak or having any psychological problem.

### Survey instrument and data collection

Questionnaires were adapted from NDHS 2006 [24] and the WHO infant feeding guidelines [5, 25]. The Nepali version of questionnaire was pretesting in neighbouring VDC of the study setting and language was edited to make it understandable for mothers. Two female enumerators were trained for data collection who had higher secondary level education, and experience of working in health and nutrition programs in the local communities. Face to face interviews were conducted with eligible mothers.

The outcome variables of this study were based on core infant indicators mentioned in the infant feeding guideline developed by the WHO [5]. Timely initiation of complementary feeding: referred to the initiation of complementary foods to children at 6–8 months of age [5, 25]. Minimum acceptable diet was defined as the child receiving at least the minimum dietary diversity and the minimum meal frequency in the last 24 h at the time of survey. For non-breastfed children, minimum dietary diversity was calculated by excluding milk feeds from the diversity score. Minimum meal frequency was defined as the number of times the child was fed based on age requirement in addition to breast feeding. Minimum dietary diversity was based on the consumption of foods from at least four food groups from the following seven food groups on the previous day (last 24 h) [5, 24]: (i) grains, roots, tubers (ii) legumes and nuts (iii) dairy products (milk, yogurt, cheese) (iv) flesh foods (meat, poultry and liver/organ meats) (v) eggs (vi) vitamin A rich fruits and vegetables; and (vii) other fruits and vegetables.

The independent variables of this study were selected based on the literature review of similar studies done in different settings [12, 17–19]. Ethnicity of mother was reported based on the Health Management Information System (HMIS), used by Ministry of Health, Government of Nepal [26]. Wealth ranking was based on the principal component analysis using household assets: type of toilet facility, type of fuel mainly used for cooking, separate kitchen for cooking, materials used for roof, floor and wall of the house; and some of the household possessions (motor cycle, landline telephone, color TV, refrigerator, computer and heavy vehicles) [24]. A wealth score was generated then divided into five quintiles: poorest, poor, middle, rich and richest.

### Statistical analysis

The percentage of infants meeting the recommended IYCF practices was reported as percentage, mean, standard deviation (SD). Factors associated with IYCF practices were reported using multiple logistic regressions. Factors were reported to be statistically significantly associated if p value was equal to or less than 0.05. The analysis was performed with Statistical Package for Social Science IBM statistics 17.0 for Windows.

### Research ethics

Approval was taken from Institutional Review Board, Human Research Ethics Committee, Institute of Medicine, Tribhuvan University Nepal. Informed consent was obtained from mothers for themselves and their children before interview. Personal identifiers such as name and household numbers were removed before analyses to maintain confidentiality.

## Results

### Characteristics of study population

Table 1 presents the characteristics of participants. Of the 180 eligible participants, two interviews yield incomplete information and they were subsequently removed from analysis. The mean age of mothers was 25 years (SD: 4.3 years) and mean age of children was 13 months (SD: 4.8 months). About 47 % had primary level education and only 22 % had higher education. Only a small proportion (7 %) mothers were involved in income generating activities. About one-third (37 %) mothers delivered their last child at a health facility. One-fifth of mothers perceived the size of their child at birth as small. Only one-third of mothers utilized postnatal care services for at least one time after delivering their last child. Only 42 % of mothers reported that they visited health facilities or outreach clinics for growth monitoring of their children.

**Table 1:** Demographic characteristics of the study population

Characteristics	Number (n = 178)	Percentage
Ethnicity of mothers		
Dalits	23	12.9
Disadvantaged Janajati	48	27.0
Disadvantaged non-Dalit Terai (plain) caste group	55	30.9
Religious minorities	4	2.2
Relatively advantaged Janajati	11	6.2
Upper caste group	37	20.8
Age of mothers (Mean ± SD: 25.2 ± 4.3)		
<20 years	8	4.5
20–34 years	161	90.4
≥35 years	9	5.1
Size of household (Mean ± SD: 7.7 ± 3.6)		
≤6	83	46.6
>6	95	53.4
Type of the family		
Nuclear	48	27.0
Joint	130	73.0
Number of children in the family		
Single child	73	41.0
≥2	105	59.0
Birth interval in months (n = 105)		
<24	12	11.4
≥24	93	88.6
Sex of the index child		
Female	81	45.5
Male	97	54.5
Age of the index child (Mean ± SD: 13.4 ± 4.8)		
6–8 months	34	19.1
9–11 months	38	21.3
12–23 months	106	59.6
Mother’s occupation		
Home maker	157	88.2
Small scale business	5	2.8
Service	7	3.9
Agriculture	9	5.1
Mother’s education		
Up to primary level	83	46.6
Some secondary	56	31.5
SLC and above	39	21.9
Earning status of mother		
Earning	12	6.7
Not earning	166	93.3
Wealth rank		
Lower	88	49.4
Higher	90	50.6
Household ownership of agricultural land		
Yes	117	65.7
No	61	34.3
Duration of food sufficiency (n = 117)		
≤6 months	31	26.5
≤12 months	66	56.4
>12 months	20	17.1
Decision makers on child feeding		
Mother	145	81.5
Father	3	1.7
Grandmother	27	15.2
Other	3	1.7
Work load of mothers (Mean ± SD: 5.9 ± 2.2)		
<8 h	135	75.8
≥8 h	43	24.2
Place of delivery		
Health facility	66	37.1
Other than health facility (home)	112	62.9
Assistance during delivery (n = 112)		
Trained health worker	2	1.1
Relative	70	39.3
Self-delivery	40	22.5
Perceived size at birth		
Normal	141	79.2
Small	37	20.8
PNC service		
Yes	59	33.1
No	119	66.9
Childhood illness in past 30 days		
Yes	57	32.0
No	121	68.0
Place of treatment of childhood illness		
Health facility	33	18.5
Pharmacy	134	75.3
Traditional healer	11	6.2
Growth monitoring practice		
Yes	75	42.1
No	103	57.9
Timely initiation of CF		
Yes	102	57.3
No	76	42.7
Minimum dietary diversity		
Yes	63	35.4
No	115	64.6
Minimum meal frequency		
Yes	149	83.7
No	29	16.3
Minimum acceptable diet (MAD)		
Yes	58	32.6
No	120	67.4

### IYCF practices and their associated factors

A total of 102 (57 %) were introduced complementary food by 6–8 months, 149 (84 %) received the recommended minimum meal frequency, 63 (35 %) received the recommended minimum dietary diversity and only 58 (33 %) received the recommended minimum acceptable diet (Table 1).

Table 2 presents the results of the association of timely initiation of complementary feeding with independent variables. Mothers who attained secondary or higher education were more likely [adjusted Odds Ratio (aOR): 2.10; 95 % CI (1.01–3.94)] to introduce complementary feeds on time. Similarly, mothers with lower workload were also more likely [aOR: 2.11; 95 % CI (1.01–4.42)] to provide complementary feeding on time.

**Table 2:** Factors associated with timely initiation of complementary feeding

Characteristics	Unadjusted OR (95 % CI)	p value	Adjusted OR (95 % CI)	p value
Ethnicity				
Disadvantaged group	1		1	
Advantaged group	1.945 (0.965–3.920)	0.061	1.248 (0.560–2.777)	0.588
Growth monitoring practice				
No	1		1	
Yes	2.397 (1.283–4.476)	0.006	1.862 (0.916–3.787)	0.086
Mother’s education				
Up to primary level	1		1	
Secondary and above	2.694 (1.461–4.967)	0.001	1.998 (1.013–3.941)*	0.046
Workload of mother in hours				
≥8	1		1	
<8	2.016 (1.007–4.038)	0.046	2.116 (1.013–4.419)*	0.046

* Significant at p < 0.05

Table 3 presents the results of association of minimum acceptable diets with independent variables. It was found that the mothers who attained high school or higher education were more likely [aOR: 3.02; 95 % CI (1.318–6.98)] to provide minimum acceptable diets to their children in the last 24 h than their counterparts with lower level of education. Mothers who took their children for growth monitoring were more likely [aOR 2.15; 95 % CI (1.02–4.54)] to provide recommended minimum acceptable diet in the past 24 h.

**Table 3:** Factors associated with minimum acceptable diets

Characteristics	Unadjusted OR (95 % CI)	p value	Adjusted OR (95 % CI)	p value
Number of children				
≥2	1		1	
Single child	1.727 (0.916–3.258)	0.090	1.109 (0.535–2.299)	0.781
Family type				
Nuclear	1		1	
Joint	1.905 (0.889–4.082)	0.094	2.016 (0.858–4.735)	0.108
Ethnicity				
Disadvantaged group	1		1	
Advantaged group	2.824 (1.419–5.617)	0.003	1.626 (0.727–3.634)	0.236
Growth monitoring practice				
No	1		1	
Yes	3.399 (1.768–6.534)	<0.001	2.149 (1.016–4.545)*	0.045
Mother’s education				
Up to primary level	1		1	
Secondary and above	4.846 (2.368–9.916)	<0.001	3.023 (1.308–6.985)*	0.010
Wealth rank				
Lower	1		1	
Higher	1.798 (0.951–3.400)	0.070	1.075 (0.519–2.226)	0.845

* Significant at p < 0.05

## Discussion

This study found that the introduction of complementary feeding to infants after 6 months was 57 % whereas minimum meal frequency is high (84 %) [27]. The current study reported that one-third of the children were suffering from diarrhoea or fever in the past 30 days, but not post 24 h at time of interview. Therefore possible effects of illness on feeding practices were not taken into considerations. A review of studies conducted in South Asia in 2016 reported that the recommended IYCF practices were less during diarrhoea because of poor appetite (perceived or real). Similarly, the proportion of infants getting recommended meal diversity (35 %) and minimum acceptable diet (33 %) is much lower. A study in South Asian countries reported that the children of 6–23 months had received minimum dietary diversity (82 %), India (15 %), Sri Lanka (71 %). However, majority of infant and young children in our study setting did not meet the recommended feeding practices. This finding is inconsistent with Bangladeshi study which showed that food items are present at household and diversity of required food can be met at local level [17]. It may be due to the fact that the majority of the community depends on specific staple foods available at the local level such as rice, wheat, potato. Even though children are fed with adequate frequency, food items remain the same with poor diversity. Moreover, in Nepalese context, there is widespread cultural belief of cereal foods having high energy contents would be enough for child growth, thereby ignoring the importance food diversity.

Studies from Asian countries showed that mother’s education was significantly associated with infant feeding practices; timely initiation of complementary food and minimum acceptable diet. Maternal education has been found to be positively associated with infant feeding in other studies [12, 14, 28] and the association is consistent in our study. This might be due to the inability of illiterate mothers to read health education materials provided while visiting health facilities. A recent study on early initiation of breastfeeding from the Nepal Demographic and Health Surveys highlighted that maternal education has positive impact on early initiation [28, 29]. This study further adds that the benefit is not limited to early infancy but also goes beyond infancy. Similar information was reported with breastfeeding messages [13].

The workload of mothers was another important factor that affected timely initiation of complementary feeding. Increasing workload is a challenge for mothers to initiate and sustain proper infant feeding practices [18, 30]. Nepali women are culturally and traditionally expected to be responsible for infant feeding, preparation of meals for the entire family and all household chores [15, 31]. Anecdotal evidence show that women work more than 16 h a day in rural and semi-urban areas due to their household chores. Such burdens provide little time for them to spend time with their young children and practice recommended infant feeds.

Growth monitoring is conducted in every health facility according to the national nutrition program, and each month 3–5 sessions of primary health care outreach clinics are carried out in each VDC [32]. In these outreach clinics, rural health workers monitor the weight of children using growth charts, and provide nutrition education to mothers or caregivers of children. These activities also provide an opportunity to early recognition of signs of under nutrition, any illness and manage them accordingly. We found that such practices were positively associated with timely initiation of the complementary food. The positive effect of such visits was also reported from Vietnam [33].

The two important findings of our study are the association of mother’s education, and regular growth monitoring with IYCF practices. This study is one of few studies conducted in the western plains of Nepal and that which followed the WHO recommended guideline on data collection and reporting. This study also has some important limitations. Due to the small sample size, the findings might not be applicable to each community of the country. Due to the cross-sectional nature of this study, conclusions on the cause-effect relationship cannot be drawn. The effect of seasonal variation and cultural practices on food availability and food consumption pattern were not taken into consideration. However, being a community-based study, this study provides an insight into infant feeding practices of Western part of Nepal.

## Conclusion

This study reported only one-third of infants met the recommended meal diversity and acceptable diet showing a major gap in infant and young child feeding practices in Western Nepal. Under-nutrition has been a major problem in Nepal and can be further complicated with poor infant feeding practices. Further programs incorporating infant feeding guidelines in health workers training manuals and more focus on educating mothers and care givers may improve infant and young child feeding practices.
